# Protective Effect of Epigallocatechin-3-Gallate in Hydrogen Peroxide-Induced Oxidative Damage in Chicken Lymphocytes

**DOI:** 10.1155/2020/7386239

**Published:** 2020-12-31

**Authors:** Xiaoqing Chi, Xiaodan Ma, Zoushuyi Li, Yong Zhang, Yong Wang, Lijia Yuan, Ye Wu, Wei Xu, Songhua Hu

**Affiliations:** Department of Veterinary Medicine, College of Animal Sci., Zhejiang University, Hangzhou, Zhejiang 310058, China

## Abstract

Epigallocatechin-3-gallate (EGCG) is one of the fundamental compounds in green tea. The present study was to evaluate the protective effect of EGCG in oxidative damage and apoptosis induced by hydrogen peroxide (H_2_O_2_) in chicken lymphocytes. Results showed that preincubation of lymphocytes with EGCG significantly decreased H_2_O_2_-reduced cell viability and apoptotic cells with DNA damage, restored the H_2_O_2_-dependent reduction in total antioxidant capacity (T-AOC), glutathione peroxidase (GSH-PX), superoxide dismutase (SOD), glutathione (GSH), and glutathione disulfide (GSSG), and suppressed the increase in intracellular reactive oxygen species (ROS), nitric oxide (NO), nitric oxide synthesis (NOS), malondialdehyde (MDA), lipid peroxide (LPO), and protein carbonyl (Carbonyl). In addition, preincubation of the cells with EGCG increased mitochondrial membrane potential (MMP) and reduced calcium ion ([Ca^2+^]i) load. The protective effect of EGCG in oxidative damage in lymphocytes was accompanied by mRNA expression of SOD, Heme oxygenase-1 (HO-1), Catalase (CAT), GSH-PX, nuclear factor erythroid 2-related factor 2 (Nrf2), and thioredoxin-1 (Trx-1). As EGCG had been removed before lymphocytes were challenged with H_2_O_2_, the activation of genes such as Nrf2 and Trx-1 by preincubation with EGCG could be the main reason for EGCG to protect the cells from oxidative damage by H_2_O_2._ Since oxidative stress is an important mechanism of biological damage and is regarded as the reasons of several pathologies, the present findings may be helpful for the use of tea products to prevent oxidative stress and maintain healthy in both humans and animals.

## 1. Introduction

The poultry industry has become more and more important in daily life. The consumption of global meat has increased 52% since 2001, and the proportion of chicken consumption has risen from 32% to 42% [1]. In 2014, FAO predicted that poultry production will surpass pork production and become the first in 2022 [2]. However, poultry diseases are important harmful factors that affect the animals. In the poultry industry, oxidative stress is regarded to play an important role in the pathophysiology of infectious bursal disease, Newcastle disease, avian influenza, etc. [3]. Reactive oxygen species (ROS) and free radicals generated from various stresses or physiological factors may be responsible for oxidative stress and various pathological effects, including DNA damage, mitochondrial dysfunction, and cell injury [4]. Oxidative stress can cause cell damage and excessive production of ROS or other oxygen-free radicals (e.g., ONOO^•−^, O_2_^•−^, and hydrogen peroxide) have been found to attenuate the antioxidant system in the organs such as the brain and testis, particularly in the spleen [5, 6]. It has been reported that the innate and adaptive immunity is damaged during oxidative stress [7, 8]. Suppressed immune responses caused by oxidative stress have been found in both mammalians and birds [9–11]. Therefore, the control of oxidative stress in the poultry industry may improve the bird's immunity and enhance the resistance of poultry against diseases and is of significance in veterinary practice.

**Figure 1:**
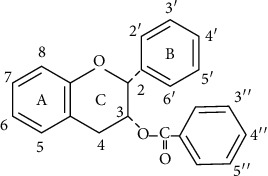
Structure of epigallocatechin-3-gallate (EGCG).

Some natural compounds have been found a tendency to quench redundant-free radicals and decrease oxidative stress [12]. Al-Sayed et al. reported that a proanthocyanidin-rich fraction obtained from *B. hookeri* (BHPF) had the hepato- and nephroprotective effect, and the fraction was able to reduce the rate of lipid peroxidation, enhance the antioxidant defense status, and guard against the pathological changes of the liver and kidney caused by oxidative damage [13]. A previous study showed that oral administration of tea extract made from green tea, the leaf of a plant *Camellia sinensis* (L) O. Ktze., had an effect in oxidative stress induced by cyclophosphamide in chickens. Green tea contains large amounts of tea catechins with antioxidant properties [14]. Epigallocatechin-3-gallate (EGCG) (Figure 1), epigallocatechin (EGC), epicatechin-3-gallate (ECG), and epicatechin (EC) are the main components [15]; meanwhile, EGCG rates 50-80% of total catechins [16]. Katiyar et al. highlighted that the EGCG could be a topical agent for ultimate usefulness against ROS-associated inflammatory dermatosis, photoaging, and photocarcinogenesis induced by UVB [17].

Because of the rich natural resource of tea, it has become possible to use the leaf and its products as an antioxidant agent in poultry production. Although numerous studies have been reported on the antioxidative activities of tea, no study has been found regarding the mechanisms underlying its effect in oxidative stress in chickens.

The present study was designed to investigate the effect of EGCG against oxidative stress of chicken lymphocytes induced by hydrogen peroxide (H_2_O_2_) with the goal of further understanding of tea and its products as an antioxidant agent in chicken production.

## 2. Materials and Methods

### 2.1. Chemicals

Epigallocatechin-3-gallate (EGCG) (93894), 3-(4, 5-dimethyl-2-thiazolyl)-2, 5-diphenyl-2-H-tetrazolium bromide (MTT) (M2128), and 34.5-36.5% H_2_O_2_ (18304) were the products of Sigma-Aldrich Inc. (St. Louis, Missouri, USA); phosphate-buffered saline (PBS) (10010023) and RPMI-1640 medium (7200047) were the products of Gibco (Thermo Fisher Scientific, Waltham, USA); lymphocyte separation medium (P9120) and cell lysis buffer (R0030) were the products of Solarbio Co. Ltd. (Beijing, China); fetal bovine serum (FBS) (13011-8611) was the product of Sijiqing Co. (Hangzhou, China).

### 2.2. Reagent Kits

Coomassie Brilliant Blue (CBB) kit (A045-2-2) and detection kits for total antioxidant capacity (T-AOC) (A015-2-1), glutathione peroxidase (GSH-PX) (A005-1-2), nitric oxide (NO) (A013-2-1), nitric oxide synthesis (NOS) (A014-2-2), malondialdehyde (MDA) (A003-4-1), lipid peroxide (LPO) (A106-1-3), and protein carbonyl (Carbonyl) (A087-1-2) were the products of Nanjing Jiancheng Institute of Bioengineering and Technology (Nanjing, China); detection kits for glutathione (GSH), glutathione disulfide (GSSG) (G263), and DNA damage quantification kit (DK02) were the products of Dojindo Molecular Technologies (Rockville, MD, USA); assay kits for reactive oxygen species (ROS) and superoxide dismutase (SOD) (ab139476), genomic DNA extraction kit (ab156900), and intracellular calcium ion ([Ca^2+^]i) detection kit (Fluo-3/AM) (ab145254) were the products of Abcam (Cambridge, UK); apoptosis detection kit (556547) was the product of BD Biosciences (San Jose, CA, USA); mitochondrial membrane potential (MMP) detection kit (JC-1) (M8650) was the product of Solarbio Co. (Beijing, China); RNAiso™ Plus kit (9108) and PrimeScript™ RT reagent kit (6210A) were the products of Takara (Dalian, China).

### 2.3. Lymphocytes

The procedures on handling animals in this experiment were approved by the Institutional Animal Care and Use Committee of Zhejiang University. Lymphocytes were isolated from chickens as described by Bi et al. [18]. Briefly, the spleens were collected from 20 d old female Sanhuang chickens (Ningbo Zhenning Stock Breeding Inc., Ningbo, China) and polished to homogenate. Cell suspension was obtained by gently pushing the homogenate through a 70 *μ*m sterile plastic mesh. The suspension was centrifuged at 2,000 × g for 15 min at room temperature on an equivalent volume of lymphocyte separation medium (1.085-1.092 g/mL). White cells in the interface were collected and washed. Cell density was adjusted to 5 × 10^5^ cells/mL and cultured in RPMI 1640 containing HEPES and 2 mM glutamine, 100 units of penicillin/mL, and 100 *μ*g of streptomycin/mL, supplemented with 10% FBS at 39°C in a humidified atmosphere containing 5% CO_2_. The viable cells were more than 95% by trypan blue exclusion test, and the lymphocyte proportion was more than 85%.

### 2.4. Treatment of Cells with EGCG and H_2_O_2_

To each well of a 12-well culture plate, 1 mL of cell suspension (5 × 10^5^ cells) was added first, and EGCG was then added at the final concentrations of 90, 45, 22.5, or 0 *μ*M. The mixtures were incubated at 39°C in a humidified atmosphere in 5% CO_2_ for 6 h. Afterward, cells were washed in PBS twice to remove EGCG and incubated in a media containing H_2_O_2_ (80 *μ*M) for an additional 4 h. Cells without treatment of EGCG and H_2_O_2_ were used as normal control or treated with H_2_O_2_ only were served as models (Table 1). After centrifuging the plates at 1,000 × g for 10 min, the supernatant was discarded and the cells were resuspended in PBS with a density of 5 × 10^5^ cells/mL for determination of cell viability, intracellular enzymes and redox parameters, apoptosis, mitochondrial membrane potential (MMP), [Ca^2+^]i concentration, and mRNA expression of SOD, GSH-PX, heme oxygenase-1 (HO-1), catalase (CAT), nuclear factor erythroid 2-related factor 2 (Nrf2), and thioredoxin-1 (Trx-1).

### 2.5. Cell Viability

The cell viability was measured by the MTT method [19]. Briefly, 100 *μ*L of cell suspension (5 × 10^4^ cells) was added to 96-well culture plates. Then, 50 *μ*L of MTT solution (2 mg/mL) was added and incubated for 4 h. After that, the plates were centrifuged at 1,000 × g for 10 min; untransformed MTT in supernatant was carefully removed. The MTT formazan was dissolved by adding 150 *μ*L of DMSO (0.04 N HCl). The optical density (OD) was read at 570 nm in Multiskan FC (Thermo, USA). The well was set in triplicate.

### 2.6. Assay for Intracellular Antioxidant Enzymes and Redox Products

To measure intracellular antioxidant enzymes and redox products, lymphocytes (5 × 10^5^ cells) were lysed by a lysis buffer to release the intracellular content for biochemical analysis. Protein concentration was determined by the Coomassie Brilliant Blue (CBB) kit with bovine serum albumin (BSA) as standard. The levels of T-AOC, GSH-PX, NO, NOS, MDA, LPO, and Carbonyl were estimated according to the manufacturers' instructions by spectrophotometry using kits obtained from Nanjing Jiancheng Institute of Bioengineering and Technology and expressed as per milligram protein (mgprot). The activities of GSH and GSSG were measured using GSSH and GSH Quantification kits acquired from Dojindo Molecular Technologies according to the manufacturers' instructions and expressed as micromole per liter (*μ*mol/L). In brief, the assay is based on the glutathione-dependent reduction of 5,5′-dithiobis-2-nitrobenzoic acid to 5-mercapto-2-nitrobenzoic acid (𝜆_max_: 415 nm) [20–28].

### 2.7. Assay for Intracellular ROS and SOD

To measure intracellular ROS and SOD, 500 *μ*L of cell suspension (2.5 × 10^5^ cells) was mixed with 10 *μ*L ROS and 10 *μ*L SOD detection reagents in a flow tube and incubated under 37°C for 30 min in dark with periodic shakings. The ROS/SOD detection kit includes two fluorescent dyes, the green dye is for ROS detection, and the orange dye is for SOD detection. The fluorescent colors can be visualized by flow cytometry. In this study, signals resulting from ROS and SOD were reflected in the FL1 and FL2 channels, respectively, and detected by FCM (BD FACSCalibur).

### 2.8. Detection of Cell Damage

The ab156900 genomic DNA extraction kit was used for the extraction of genomic DNA from the cells according to previously described by Kuechler et al. [29]. Each sample was adjusted to contain 100 *μ*g of extracted DNA per milliliter (/mL). Oxidative attack on the deoxyribose moiety by hydroxy radical will lead to the release of free bases from DNA, generating strand breaks with various sugar modifications and abasic sites (AP). AP sites are one of the major types of damage generated by ROS. Aldehyde Reactive Probe (ARP) reagent (N′-aminooxymethylcarbonylhydrazino-D-biotin) reacts specifically with an aldehyde group which is an open ring form of the AP sites. After treatment of DNA containing AP sites with ARP reagent, AP sites are tagged with biotin residues. All AP sites will be converted to biotin-tagged forms by an excess amount of ARP. Therefore, AP sites can be quantified using avidin-biotin assay. The number of AP sites was measured using a DNA damage quantification kit based on calorimetric assay as reported [30]. Briefly, 10 *μ*L of DNA extract and an equal volume of ARP solution was mixed in a 0.5 mL vial and incubated at 37°C for 1 h. After dilution of ARP-labeled genomic DNA with 310 *μ*L of buffer, 60 *μ*L of the diluted solution was taken to mix with 60 *μ*L of standard ARP-DNA solution in 96-well plates. Then, 100 *μ*L of DNA binding solution was added and mixed well. The plate was kept at room temperature (25°C) overnight. After DNA binding solution was discarded, the plate was washed with PBS and added with diluted 150 *μ*L of HRP-streptavidin solution. After incubation of the plate at 37°C for 1 h, 100 *μ*L of substrate solution was added and incubated at 37°C for another 1 h. The optical density (OD) was read at 650 nm. The number of AP sites in the genomic DNA was calculated according to the linear calibration curve generated for each experiment using ARP-DNA standard solutions.

### 2.9. Apoptosis Detection

An apoptosis detection kit was used to measure apoptosis of the cells as previously described [31]. Cells were washed in PBS at 1,000 × g for 10 min and resuspended in 1 mL of 1× binding buffer with adjusting to a concentration of 5 × 10^5^ cells/mL. Then, 100 *μ*L of the cell suspension was mixed with 7 *μ*L annexin V-FITC and 7 *μ*L propidium iodide (PI) staining solution and incubated for 20 min at 37°C in dark. Afterward, 300 *μ*L of 1× binding buffer was added, and fluorescence in cells was detected by FCM. The data were analyzed using FlowJo V10 software, and apoptotic rate was expressed as the ratio of apoptotic cells to the total number of cells.

### 2.10. Measurement of Mitochondrial Membrane Potential (MMP)

Mitochondrial membrane potential (MMP) of the lymphocytes was measured using 5,5′,6,6′-tetrachloro-1,1′,3,3′-tetraethyl-imidacarbocyanine iodide (JC-1) by flow cytometry as previously described [32]. Briefly, cells (2.5 × 10^5^ cells) were cultured in 0.5 mL of medium containing JC-1 (5 *μ*g/mL) in dark for 30 min at 37°C with periodic shaking to ensure a beneficial suspension. After washing by centrifugation at 1,000 × g for 10 min in staining buffer, the cells were resuspended in 0.5 mL of PBS and then assayed in FCM within 1 h. The data were analyzed using FlowJo V10 software. The reduction of the red/green ratio illustrates decreased MMP, which is presented by the polymer mean fluorescent intensity (MFI)/monomer MFI.

### 2.11. Assay for Intracellular Calcium Ion ([Ca^2+^]i)

Fluo-3/AM fluorescence density was measured to determine intracellular [Ca^2+^]i concentration as previously described [33]. In brief, 10 *μ*L of Fluo-3/AM was added into 1 mL of cell suspensions (5 × 10^5^ cells) and incubated at 37°C for 45 min in dark. After washing, the cells were suspended in PBS, and Fluo-3/AM fluorescent intensity was measured by flow cytometry. Data were analyzed using FlowJo V10 software and expressed as MFI of Fluo-3/AM.

### 2.12. Real-Time Quantitative PCR Analysis

Total RNA was extracted from lymphocytes (5 × 10^5^ cells) using RNAiso™ Plus kit according to the manufacturer's protocol. The purity and quality of total RNA were evaluated by the ratio of absorption (260 : 280 nm) ranged from 1.8 to 2.0 and quantitated on a NanoPhotometer spectrophotometer (IMPLEN, CA, USA) [34]. Subsequently, 2 *μ*g of total RNA was used to synthesize cDNA using a PrimeScript™ RT reagent kit based on the manufacturer's instructions. The primers for RT-qPCR analysis were listed in Table 2. *β*-Actin was used as an internal control to normalize gene expression. Primers were designed by Primer 5 software and synthesized by Takara (Takara, Dalian, China). For RT-qPCR reactions, 20 *μ*L mixtures were made up, containing 2 *μ*L cDNA, 10 *μ*L SYBR Taq, 1.6 *μ*L forward and reverse primer (1 : 1), 0.4 *μ*L ROX Reference Dye, and 6 *μ*L RNase-free water. Each sample was set with triplicate. SOD, HO-1, CAT, GSH-PX, Nrf2, and Trx-1 expression of messenger RNA (mRNA) were detected by performing RT-qPCR reactions on Bio-Rad T100 (Bio-Rad Laboratories, Inc., USA). The PCR procedure included 95°C for 2 min, followed by 35 cycles of 95°C for 10 s, 58°C for 30 s, and 72°C for 30 s. Only one peak for each PCR product was analyzed by melting curve. The comparative Ct value method was used to quantify mRNA expression relative to *β*-actin expression using the 2^-*ΔΔ*CT^ method. The relative mRNA expressions of SOD, HO-1, CAT, GSH-PX, Nrf2, and Trx-1 were expressed as mean ± standard deviation (S.D.).

### 2.13. Statistical Analysis

One-way ANOVA of SPSS Statistics for Mac (Version 20) was used for statistical analysis of the data, followed by Tukey's post hoc test for multiple comparisons between groups. The results were presented as the means ± S.D. All statistical analyses were performed using the GraphPad InStat software (Version 7, GraphPad). *P* value < 0.05 or *P* value < 0.01 was considered statistically significant.

## 3. Results

### 3.1. Effect of EGCG on Cell Viability

**Figure 2:**
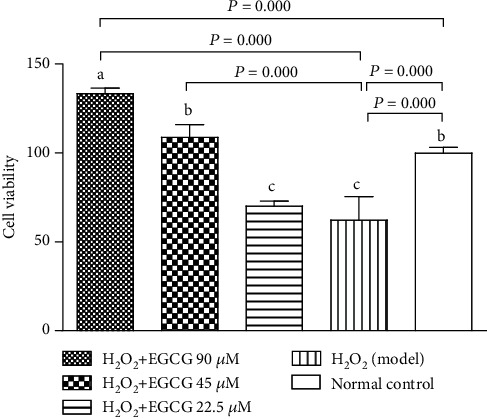
Effect of EGCG on cell viability. Lymphocytes (5 × 10^4^) were treated with EGCG (0, 22.5, 45, and 90 *μ*M) for 6 h first and then cultured in media with (model) or without (normal control) H_2_O_2_ (80 *μ*M) for additional 4 h. Cell viability was determined by the MTT assay using the absorbance of formazan at 570 nm. Data are presented as % of the normal control (mean ± S.D., *n* = 6). Bars with different letters are significantly different (*P* < 0.05 or *P* < 0.01).

To evaluate the effect of EGCG on cell viability, lymphocytes were cultured with EGCG first and then treated with H_2_O_2_ to induce oxidative stress of the cells. Cell viability was determined by the MTT assay.  Figure 2 showed that the number of viable cells significantly decreased after lymphocytes were treated with H_2_O_2_ (model) when compared to the normal control (*P* < 0.01). However, pretreatment of the cells with EGCG increased the number of viable cells in a dose-dependent manner at the range of 22.5-90 *μ*M when compared to the model group.

### 3.2. EGCC Increased Antioxidant Capacity and Decreased Redox Products

**Figure 3:**
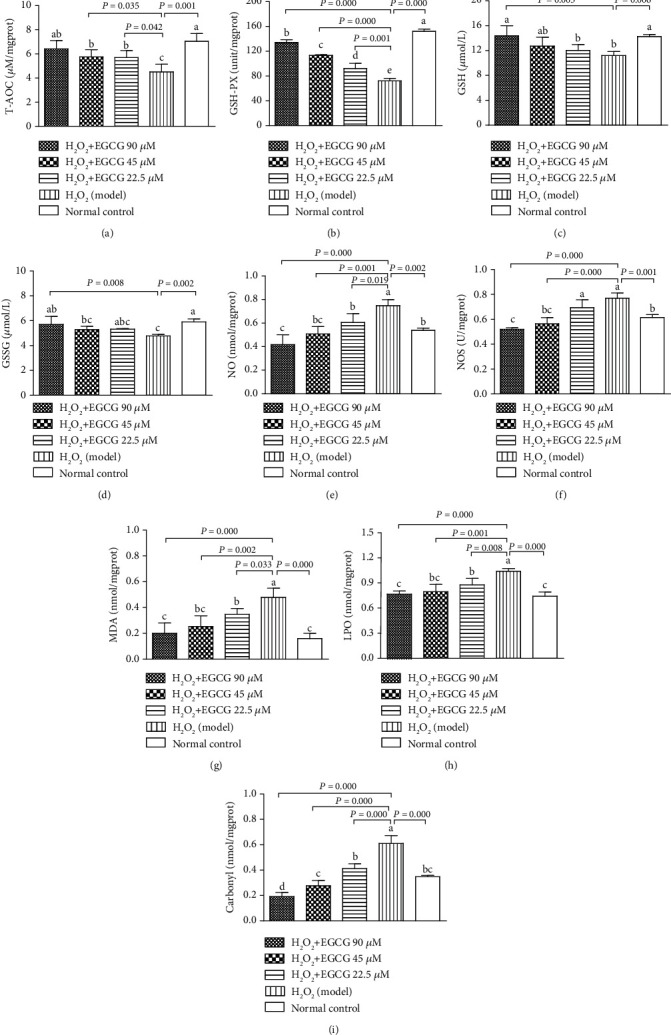
Intracellular antioxidants and redox products. Lymphocytes (5 × 10^5^) were treated with EGCG (0, 22.5, 45, and 90 *μ*M) for 6 h first and then cultured in media with (model) or without (normal control) H_2_O_2_ (80 *μ*M) for an additional 4 h. The cells were treated in lysis buffer to release the intracellular materials for analysis of enzymatic (T-AOC and GSH-PX) and nonenzymatic (GSH and GSSG) antioxidants (a–d) and redox products (NO, NOS, MDA, LPO, and Carbonyl) (e–i). Data are presented as mean ± S.D. (*n* = 6). Bars with different letters are significantly different (*P* < 0.05 or *P* < 0.01).

To observe the redox state in the lymphocytes, intracellular contents released from the cells were analyzed. Figures 3(a)–3(d) showed that incubation of the lymphocytes with H_2_O_2_ significantly suppressed enzymatic (T-AOC and GSH-PX) and nonenzymatic (GSH and GSSG) antioxidant capacity; and preincubation with EGCG significantly enhanced the antioxidant ability when compared to the model group, indicating that pretreatment of lymphocytes with EGCG prevented H_2_O_2_-induced suppression in antioxidant capacity in cells. Figures 3(e)–3(i) showed that redox products (NO, NOS, MDA, LPO, and Carbonyl) were significantly increased after incubation of the lymphocytes with H_2_O_2_ but preincubation with EGCG significantly decreased the products, suggesting that pretreatment of the lymphocytes with EGCG could prevent the increased production of redox products due to decreased antioxidant capacity induced by H_2_O_2_.

### 3.3. EGCC Decreased ROS and Increased SOD Generation

**Figure 4:**
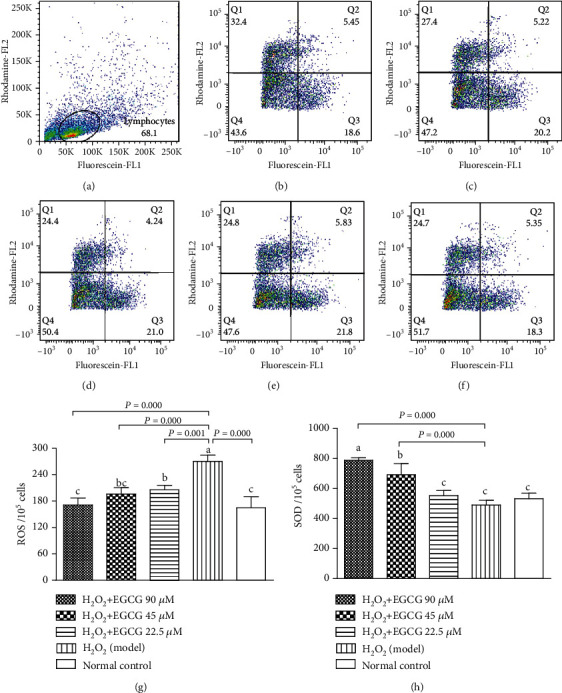
Intracellular ROS and SOD. Lymphocytes (2.5 × 10^5^) were treated with EGCG (0, 22.5, 45, and 90 *μ*M) for 6 h first and then cultured in media with (model) or without (normal control) H_2_O_2_ (80 *μ*M) for 4 h, then dyed for ROS and SOD. Cells with increased production of SOD showed orange fluorescence and were detected with the FL2 channel. Cells with increased production of ROS demonstrated green light and were registered in the FL1 channel. (a) Flow cytometric gating percentage; (b) H_2_O_2_ + EGCG 90 *μ*M group; (c) H_2_O_2_ + EGCG 45 *μ*M group; (d) H_2_O_2_ + EGCG 22.5 *μ*M group; (e) H_2_O_2_ (model) group; (f) normal control group; (g) ROS levels represented by mean green fluorescence intensity in the indicated groups; (h) SOD levels represented by mean orange fluorescence intensity in the indicated groups. Data are presented as mean ± S.D. (*n* = 6). Bars with different letters are significantly different (*P* < 0.05 or *P* < 0.01).

To observe intracellular ROS and SOD, lymphocytes were dyed with a ROS/SOD detection kit and analyzed by FCM.  Figure 4 showed that intracellular ROS significantly increased and SOD numerically decreased after incubation of the lymphocytes with H_2_O_2_ when compared to the normal control. However, preincubation of the lymphocytes with EGCG significantly decreased ROS and increased SOD when compared to the model. The results indicated that pretreatment of the lymphocytes prevented the intracellular production of reactive oxygen species (ROS) and enhanced the capacity of superoxide dismutase (SOD) in cells in stress induced by H_2_O_2_.

### 3.4. EGCC Decreased Cell Damage and Apoptosis

**Figure 5:**
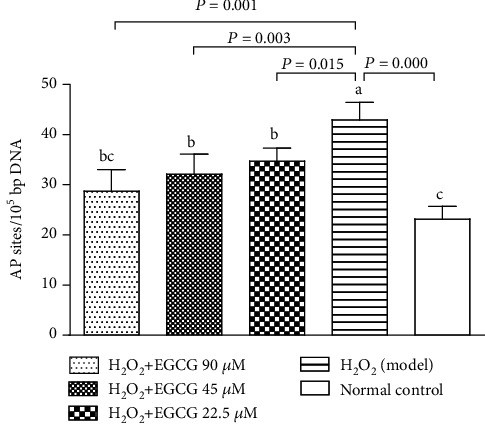
Effect of EGCG on cell DNA damage. Lymphocytes (5 × 10^5^) were treated with EGCG (0, 22.5, 45, and 90 *μ*M) for 6 h first and then cultured in media with (model) or without (normal control) H_2_O_2_ (80 *μ*M) for an additional 4 h. The oxidative DNA damage in cells was estimated with apurinic/apyrimidinic sites (AP sites). The number of AP sites was measured using a DNA damage quantification kit based on calorimetric assay. Data are presented as mean ± S.D. (*n* = 6). Bars with different letters are significantly different (*P* < 0.05 or *P* < 0.01).

**Figure 6:**
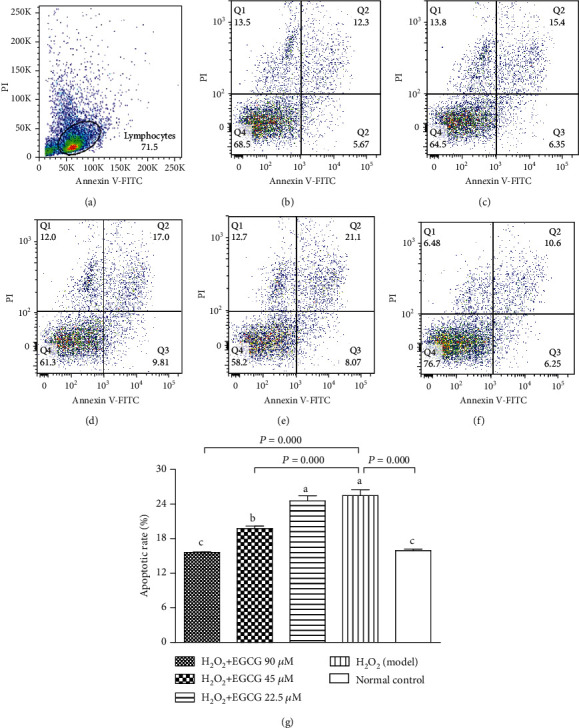
Effect of EGCG on cell apoptosis. Lymphocytes (5 × 10^5^) were treated with EGCG (0, 22.5, 45, and 90 *μ*M) for 6 h first and then cultured in media with (model) or without (normal control) H_2_O_2_ (80 *μ*M) for an additional 4 h. Apoptotic cells (annexin V+/PI-) were discriminated with FCM analysis. (a) Flow cytometric gating percentage; (b) H_2_O_2_ + EGCG 90 *μ*M group; (c) H_2_O_2_ + EGCG 45 *μ*M group; (d) H_2_O_2_ + EGCG 22.5 *μ*M group; (e) H_2_O_2_ (model) group; (f) normal control group; (g) the ratio of apoptotic cells to the total number of cells in the indicated groups. Values are expressed as mean ± S.D. (*n* = 6). Bars with different letters are significantly different (*P* < 0.05 or *P* < 0.01).

In this study, the number of apurinic/apyrimidinic sites (AP sites) was used to estimate cell DNA damage, and FCM analysis was used to detect apoptotic cells (Annexin V+/PI-). Figures 5 and 6 showed that incubation of the lymphocytes with H_2_O_2_ significantly increased the number of the AP sites and apoptotic cells; preincubation of cells with EGCG significantly decreased the number of the AP sites and apoptotic cells. The results suggested that pretreatment with EGCG prevented cell damage and apoptosis caused by H_2_O_2_ treatment.

### 3.5. EGCC Increased Mitochondrial Membrane Potential and Decreased [Ca^2+^]i

**Figure 7:**
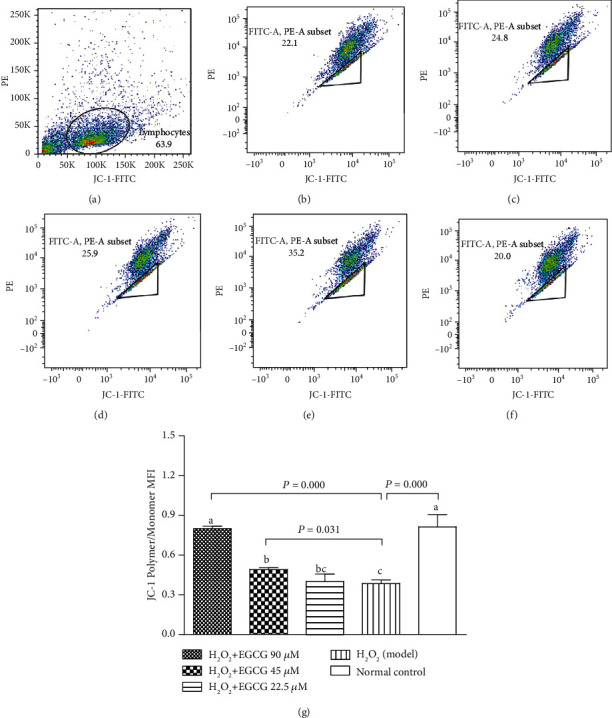
Effect of EGCG on mitochondrial depolarization. Lymphocytes (2.5 × 10^5^) were treated with EGCG (0, 22.5, 45, and 90 *μ*M) for 6 h first and then cultured in media with (model) or without (normal control) H_2_O_2_ (80 *μ*M) for an additional 4 h. Cells were incubated with JC-1 (5 *μ*g/mL) and assayed by FCM. Mitochondrial depolarization was represented by a reduction in the red/green fluorescence intensity ratio. (a) Flow cytometric gating percentage; (b) H_2_O_2_ + EGCG 90 *μ*M group; (c) H_2_O_2_ + EGCG 45 *μ*M group; (d) H_2_O_2_ + EGCG 22.5 *μ*M group; (e) H_2_O_2_ (model) group; (f) Normal Control group; (g) bar diagram represented JC-1 polymer/monomer MFI. Values are expressed as mean ± S.D. (*n* = 6). Bars with different letters are significantly different (*P* < 0.05 or *P* < 0.01).

**Figure 8:**
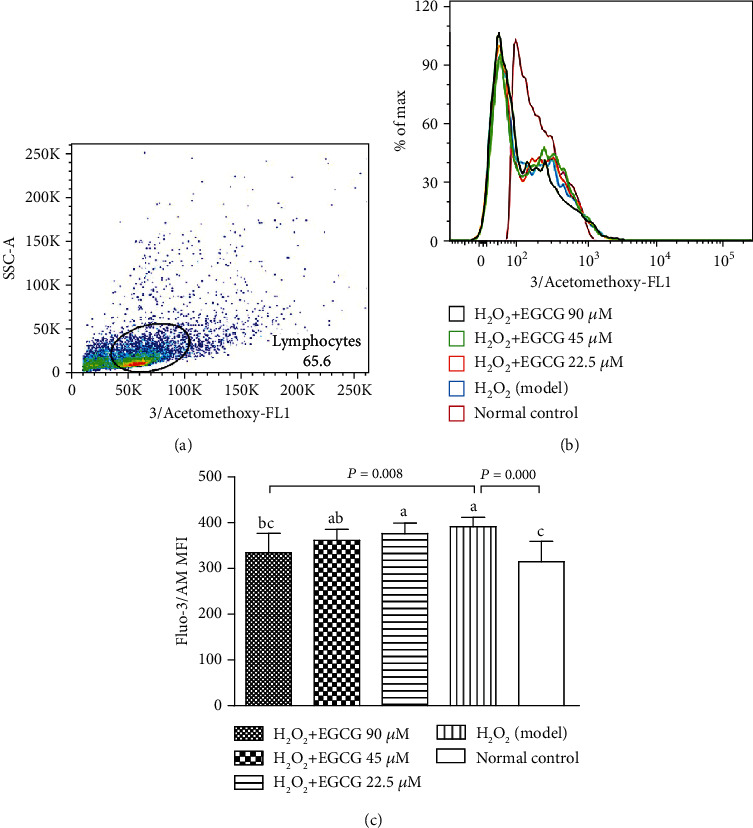
Effect of EGCG on H_2_O_2_-induced change in the [Ca^2+^]i levels. Lymphocytes (5 × 10^5^) were treated with EGCG (0, 22.5, 45, and 90 *μ*M) for 6 h first and then cultured in media with (model) or without (normal control) H_2_O_2_ (80 *μ*M) for an additional 4 h. Afterward, the cells were incubated with Fluo3/AM ([Ca^2+^]i probe), and Fluo-3/AM fluorescence was measured by FCM. (a) Flow cytometric gating percentage; (b) the fluorescence intensity of Fluo-3/AM reflected the [Ca^2+^]i levels; (c) bar diagram represented Fluo-3/AM MFI. Values are expressed as mean ± S.D. (*n* = 6). Bars with different letters are significantly different (*P* < 0.05).

To estimate the integrity of the cell membrane, mitochondrial membrane potential (MMP) was measured with JC-1, and the change of [Ca^2+^]i concentrations in cells was estimated using Fluo-3/AM. Incubation of the lymphocytes with H_2_O_2_ significantly decreased MMP (Figure 7) and increased the [Ca^2+^]i in cells (Figure 8); however, preincubation with EGCG significantly increased MMP (Figure 7) and decreased the [Ca^2+^]i in cells (Figure 8), suggesting that pretreatment with EGCG prevented cell membrane damage resulting from H_2_O_2_ treatment.

### 3.6. EGCC Increased mRNA Expression of SOD, HO-1, CAT, GSH-PX, Nrf2, and Trx-1

**Figure 9:**
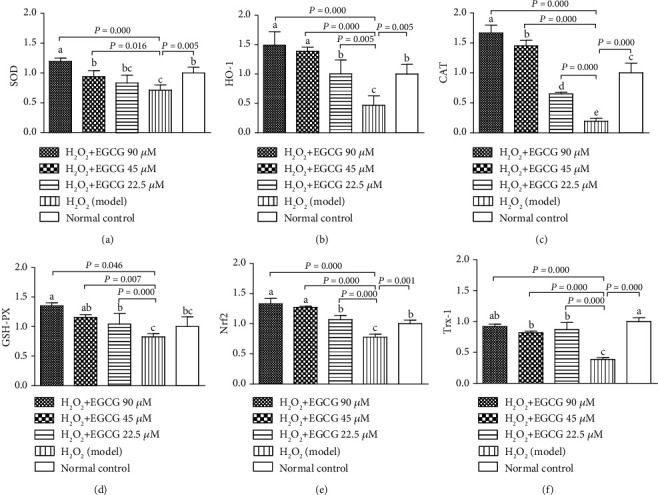
Effect of EGCG on the relative mRNA expression. Lymphocytes (5 × 10^5^) were treated with EGCG (0, 22.5, 45, and 90 *μ*M) for 6 h first and then cultured in media with (model) or without (normal control) H_2_O_2_ (80 *μ*M) for an additional 4 h. RT-qPCR was carried out to quantify mRNA of SOD, HO-1, CAT, GSH-PX, Nrf2, and Trx-1 (a–f) and data were normalized using the 2^-*ΔΔ*C^_T_ method. Values are expressed as % of the normal control (mean ± S.D., *n* = 6). Bars with different letters are significantly different (*P* < 0.05 or *P* < 0.01).

RT-qPCR was used to quantify the expression of antioxidant genes.  Figure 9 showed that incubation of the lymphocytes with H_2_O_2_ depressed the expression of mRNA of SOD, HO-1, CAT, GSH-PX, Nrf2, and Trx-1 in cells, while preincubation with EGCG significantly increased the expression of the antioxidant genes when compared to the model group, indicating that pretreatment of the cells with EGCG recovered the depressed expression of antioxidant genes caused by H_2_O_2_ treatment.

## 4. Discussion

The protective effect of EGCG in oxidative damage induced by H_2_O_2_ in lymphocytes was demonstrated. Preincubation of lymphocytes with EGCG significantly decreased H_2_O_2_-induced intracellular ROS, DNA damage, cell apoptosis, [Ca^2+^]i overload, and increased MMP, which were accompanied by the increased antioxidant capacity (T-AOC, GSH-PX, SOD, GSH, and GSSG) and decreased redox products (NO, NOS, MDA, LPO, and Carbonyl). These changes were in association with mRNA expression of SOD, HO-1, CAT, GSH-PX, Nrf2, and Trx-1.

Lymphocytes are important cells in the cellular and humoral immunity. As lymphocyte membranes are rich in unsaturated fatty acids, the cells are among the main target for oxidative stress and particularly easy to be attacked by ROS. In the poultry production, chickens are usually exposed to many harmful factors such as condensed population, polluted air, and contaminated feed or drinking water, which may cause overproduction of ROS. Increased ROS may induce oxidative stress, damage the structure of lymphocytes, and suppress the immunity. H_2_O_2_ has been usually used to induce oxidative stress in vitro. Like the process found in vivo, exogenous H_2_O_2_ crosses the cell membrane; destroys nucleic acids, proteins, and lipids; demolishes [Ca^2+^]i homeostasis; and activates mitochondria signals, ultimately leading to cell apoptosis [35–38]. In the present study, exposure of lymphocytes to H_2_O_2_ for 4 h lead to increase intracellular ROS (Figure 4), DNA damage (Figure 5), apoptotic cells (Figure 6), decrease MMP (Figure 7), and cause [Ca^2+^]i overload (Figure 8), resulting in significantly decreased cell viability (Figure 2). However, pretreatment of the cells with EGCG decreased intracellular ROS, DNA damage, apoptotic cells, and [Ca^2+^]i load and improved MMP with increased cell viability when compared to model cells. Because of the damage of lymphocytes during oxidative stress, inhibited immune responses to vaccination in association with oxidative stress were observed in chickens in our previous studies [39–41].

DNA damage under oxidative stress is a result of the interaction of DNA and ROS, mainly hydroxy radical which is converted from superoxide and hydrogen peroxide by the Fenton reaction. Hydroxy radicals cause a multiplicity of modifications in DNA. Oxidative attacks by hydroxy radicals on the deoxyribose moiety lead to the release of free bases from DNA, generating strand breaks with various sugar modifications and simple abasic sites (AP sites). AP sites are one of the major types of damage generated by ROS. The redundant ROS changes physiological homeostasis and causes tissue damage [42]. Figure 5 showed that the number of AP sites was significantly increased after H_2_O_2_ treatment but decreased after pretreatment of the cells with EGCG, indicating that EGCG reduced DNA damage caused by H_2_O_2_.

The poultry antioxidant system comprises enzymatic and nonenzymatic defenses [43, 44]. Enzymatic defenses include T-AOC, SOD, and GSH-PX. The total antioxidant capacity (T-AOC) may give more biologically relevant information than the individual antioxidants only [45]. SOD is the first line of cellular defense against oxidative damage, which converts O^2•-^ into O_2_ or H_2_O_2_, decomposes into H_2_O ultimately [46]. GSH-PX is an enzyme responsible for reducing soluble hydrogen peroxide and alkyl peroxides, scavenging ROS and its oxidation products to protect the body from lipid peroxidation [47, 48]. All these enzymes work together to eliminate active oxygen species [49]. The present study elucidated that H_2_O_2_ treatment significantly (T-AOC and GSH-PX) or numerically (SOD) depressed the enzymes, and pretreatment with EGCG neutralized the inhibitory effect of H_2_O_2_ on T-AOC, GSH-PX, and SOD as indicated in Figures 3(a) and 3(b) and 4(h). Nonenzymatic antioxidant defense includes GSH and GSSG. GSH is one of the major cellular antioxidants [50–52]. It can directly react with radicals such as superoxide and deliver electrons for the reduction of peroxides by GSH-PX [50]. The product of such reactions is GSSG, which is reduced in cells to GSH in the NADPH-dependent reaction, catalyzed by glutathione reductase [50, 52]. In the present study, GSH and GSSG were significantly decreased after the cells were treated with H_2_O_2_ and increased when the cells were pretreated with EGCG as indicated in Figures 3(c) and 3(d). Therefore, enzymatic and nonenzymatic antioxidants may cooperate and provide protection against ROS attack.

Lipid peroxidation is generated by the conversion of polyunsaturated fatty acid into lipid peroxides or MDA. As a major product of LPO, MDA is supposed to be a marker of tissue damage [53, 54]. Protein carbonyl is one of the final products of protein peroxidation, which is formed by oxidation via either an increase of ROS or the attack of reactive aldehydes such as NO, NOS, MDA, and LPO formed during lipid peroxidation [55], namely, in a way that LPO aggravates protein peroxidation. Accordingly, the protein carbonyl level is considered a surrogate marker of protein peroxidation [56]. In addition, NO is likely to cross membranes and reacts with superoxide anion which forms as a by-product of respiration to produce peroxynitrite (ONOO^•−^) and cytotoxic species that have been demonstrated to lead to lipid peroxidation as a result of [Ca^2+^]i dysfunction [57] and are theoretically conducive to enhance intracellular levels of ROS. Our study clearly showed a significant increase in NO, NOS, MDA, LPO, and Carbonyl post-H_2_O_2_ treatment, but EGCG counteracted the increase as indicated in Figures 3(e)–3(i), indicating that EGCG may effectively protect the tissue from damage by eliminating or diminishing the production of NO, NOS, MDA, LPO, or Carbonyl.

The antioxidant effect of tea in oxidative stress in chickens has been reported previously. Chi et al. observed that oral administration of tea saponins significantly increased T-AOC, SOD, CAT, GSH-PX, GSH, VC, and VE and enhanced the immune responses to Newcastle disease and infectious bronchitis vaccines in chickens with oxidative stress induced by cyclophosphamide [41]. They recently found that oral administration of tea extract granule (TEG) significantly increased T-AOC, SOD, CAT, GSH-PX, and GSH as well as decreased Carbonyl, LPO, and MDA [58]. EGCG has a characteristic structure with a large number of hydroxyl groups. The presence of an *ortho*-trihydroxyl group in the B-ring has been shown to be important to the radical scavenging abilities of EGCG; a gallate moiety at the 3 position of the C-ring increases the radical scavenging effectiveness (Figure 1) [59]. There is evidence that at least some catechin metabolites maintain comparable antioxidant capacities to their parent compounds [60]. After administration of green tea in rats reaching roughly 400 mg of catechins, measured-free catechins in the plasma were found to account for only 20% of its additive total radical-trapping parameter (TRAP), proposed that catechin conjugates and metabolites might have contributed to the measured TRAP increase [61]. These findings showed that the biological activity of tea extract may depend on its special structure, and the metabolites combined in vivo can be also with a large state role. As EGCG is the major bioactive component with its antioxidant properties, EGCG may directly contribute to the antioxidant activity when tea and its products are orally administered.

In addition, EGCG may participate in the antioxidant defense through the thioredoxin (Trx-1) system comprising of thioredoxin and thioredoxin reductase and the nuclear factor erythroid 2-related factor 2 (Nrf2) system [62]. Under abnormal conditions, the Trx-1 system serves as the first line defense system against oxidative stress, sparing the activation of the Nrf2 system [63]. Consequently, enhanced expression of the Trx-1 in the cytoplasm by directly eliminating ROS and in the nucleus by restraining the phosphorylation of I*κ*B-*α*, restraining the activation of NF-*κ*B, and making an indirect effect on antioxidation [64]. The transcription factor Nrf2 is commonly involved in the transcriptional both constitutive and inducible regulation of genes encoding antioxidant proteins under stress conditions. Some natural sources can stimulate Nrf2 signaling pathways and may be the inducer of phase II detoxifying and antioxidant enzymes representing GSH-PX and SOD [38]. SOD is an antioxidant enzyme involved in the scavenging of superoxide radicals; in the antioxidant defense system of birds, GSH-PX is more important than in the case of mammals [65], and EGCG availability can be the limiting factor for the activation of GSH-PX mRNA in chickens. In addition to inducing several phase II detoxifying enzymes, Nrf2 is also involved in the de novo synthesis of various antioxidant enzymes responsible for protection against cytotoxicity caused by oxidative stress [66]. Nrf2 promotes the expression of antioxidant enzymes (e.g., HO-1 and CAT) as a preventable response to oxidative stress. As the rate-limiting enzyme of heme decomposition reaction, HO-1 can stimulate production of endogenous carbon monoxide, biliverdin, and ferrous; biliverdin reductase converts biliverdin into bilirubin, which the two substances are possible endogenous antioxidants entailed in the structure of the defense system against ROS and free radical chain reactions (e.g., lipid peroxidation injury) [67]. Our data showed that an increased Nrf2 mRNA expression was accompanied by enhanced expression of genes SOD, CAT, GSH-PX, and HO-1 in EGCG-treated lymphocytes (Figure 8), suggesting that the activation of antioxidant genes by EGCG could mainly contribute to the reduced damage by H_2_O_2_ in EGCG-treated cells.

The present study showed in vitro antioxidant effect of EGCG, one of the fundamental compounds in tea. Tea and its products are common and popular in humans but rarely used in animals. Since oxidative stress is an important mechanism of biological damage and is regarded as the reasons of several pathologies, tea and its products may be useful to prevent oxidative stress and maintain healthy in both humans and animals.

## 5. Conclusion

The present study demonstrated the protective effect of EGCG in oxidative damage and apoptosis induced by H_2_O_2_ in chicken lymphocytes. Preincubation of lymphocytes with EGCG significantly decreased H_2_O_2_-reduced cell viability and apoptotic cells with DNA damage, restored the H_2_O_2_-dependent reduction in T-AOC, GSH-PX, SOD, GSH, and GSSG, and suppressed the increase in intracellular ROS, NO, NOS, MDA, LPO, and Carbonyl. In addition, preincubation of the cells with EGCG increased MMP and reduced [Ca^2+^]i load. The protective effect of EGCG in oxidative damage in lymphocytes was accompanied by mRNA expression of SOD, HO-1, CAT, GSH-PX, Nrf2, and Trx-1. As EGCG had been removed before lymphocytes were challenged with H_2_O_2_, the activation of genes such as Nrf2 and Trx-1 by preincubation with EGCG could be the main reason for EGCG to protect the cells from oxidative damage by H_2_O_2._

## 6. Innovation

The protective effect of EGCG was first demonstrated in oxidative damage and apoptosis induced by H_2_O_2_ in chicken lymphocytes. Since oxidative stress is an important mechanism of biological damage and is regarded as the reasons of several pathologies that affected animals, the present findings may be helpful for the use of tea products to prevent oxidative stress and maintain healthy in both humans and animals.

## Figures and Tables

**Table 1:** Experimental design.

Groups	*n*	H_2_O_2_ (*μ*M)	EGCG (*μ*M)
H_2_O_2_+EGCG 90 *μ*M	6	80	90
H_2_O_2_+EGCG 45 *μ*M	6	80	45
H_2_O_2_+EGCG 22.5 *μ*M	6	80	22.5
H_2_O_2_ (model)	6	80	0
Normal control	6	0	0

**Table 2:** Primer sequences used for real-time qPCR assay.

Gene^†^	Sequence (5′-3′)^‡^	Product size (bp)
SOD	CGTCATTCACTTCGAGCAGAAGG	233
	GTCTGAGACTCAGACCACATA	
HO-1	ACTTCTATGGCAGCAACT	129
	AATAGCGGGTGTAGGC	
CAT	CTGTTGCTGGAGAATCTGGGTC	160
	TGGCTATGGATGAAGGATGGAA	
GSH-PX	TTGTAAACATCAGGGGCAAA	140
	TGGGCCAAGATCTTTCTGTAA	
Nrf2	ATTGAGCAAGTTTGGGAGGA	214
	AAGACACTGTAACTCAGGAATGGA	
Trx-1	GCAGGACAGGCTGGAACTCACA	152
	CGAGAAGTGCGAGGTGAACG	
*β*-Actin	GAGAAATTGTGCGTGACATCA	152
	CCTGAACCTCTCATTGCCA	

^**†**^SOD: superoxide dismutase; HO-1: Heme oxygenase-1; CAT: catalase; GSH-PX: glutathione peroxidase; Nrf2: nuclear factor erythroid 2-related factor 2; Trx-1: thioredoxin-1. ^**‡**^Shown as forward primer followed by reverse primer.

## Data Availability

The data are available and acquired from the authors.
